# Honey in wound healing: An updated review

**DOI:** 10.1515/biol-2021-0084

**Published:** 2021-10-06

**Authors:** Hanaa Tashkandi

**Affiliations:** Faculty of Medicine, King Abdulaziz University, Jeddah 21589, Saudi Arabia

**Keywords:** wound healing, honey, multidrug-resistant bacteria, antibacterial effects, antifungal properties

## Abstract

Wound healing is a complex process with many interdependent pathophysiological and immunological mediators to restore the cellular integrity of damaged tissue. Cutaneous wound healing is the repair response to a multitude of pathologies induced by trauma, surgery, and burn leading to the restoration and functionality of the compromised cells. Many different methods have been employed to treat acute and chronic wounds, such as antimicrobial therapy, as most wounds are susceptible to infection from microbes and are difficult to treat. However, many antimicrobial agents have become ineffective in wound treatment due to the emergence of multiple drug-resistant bacteria, and failures in current wound treatment methods have been widely reported. For this reason, alternative therapies have been sought, one of which is the use of honey as a wound treatment agent. The use of honey has recently gained clinical popularity for possible use in wound treatment and regenerative medicine. With this high demand, a better delivery and application procedure is required, as well as research aiming at its bioactivity. Honey is a safe natural substance, effective in the inhibition of bacterial growth and the treatment of a broad range of wound types, including burns, scratches, diabetic boils (Skin abscesses associated with diabetic), malignancies, leprosy, fistulas, leg ulcers, traumatic boils, cervical and varicose ulcers, amputation, burst abdominal wounds, septic and surgical wounds, cracked nipples, and wounds in the abdominal wall. Honey comprises a wide variety of active compounds, including flavonoids, phenolic acid, organic acids, enzymes, and vitamins, that may act to improve the wound healing process. Tissue-engineered scaffolds have recently attracted a great deal of attention, and various scaffold fabrication techniques are being researched. Some incorporate honey to improve their delivery during wound treatment. Hence, the aim of this review is to summarize recent studies on the wound healing properties of honey.

## 1 Introduction

A wound is a disturbance in the normal structure and function of the epidermis. The epidermis is considered the first line of defense and protection against trauma. Various mechanisms can cause wounds, such as acute injury (abrasion, puncture, and/or crushing), surgery, and physiological conditions that compromise the skin (e.g., ischemia and pressure). Wound healing is a complex process with many interdependent immunological and pathophysiological mediators to restore the cellular integrity of the damaged tissue [1]. Wound healing depends on the presence of multiple types of cells, the extracellular matrix (ECM), cytokines, and growth factors, in addition to restoring the functionality of the compromised cells. Four distinct and overlapping stages are involved – inflammation, proliferation/regeneration, and tissue fibroplasia [1]. Recently, there has been a major increase in the burden of wound healing management due to the presence of multiple drug-resistant bacteria that can interfere with the wound repair process. Therefore, alternative natural compounds have been sought. Among those compounds is honey. The therapeutic potential of honey in the treatment of wounds and ulcers was initially recognized by the Sumerians and was known as far back as 2100–2000 BC [2]. The beneficial properties of honey have been known since ancient times [3], and its therapeutic use remained popular until the advent of antibiotics [4]. Published data show that honey benefits wound healing in the chronic inflammatory phase via the scavenging of reactive oxygen species produced by neutrophils [5,6].

With the emergence of drug-resistant bacteria, many antimicrobial agents have become ineffective in wound treatment, and many failures in current wound treatment methods have been reported. For this reason, alternative therapies have been sought, one of which is the use of honey as a wound treatment agent [7,8]. The use of honey has recently gained clinical popularity for possible use in wound treatment and in regenerative medicine.

Honey is made from the nectar of flowers collected by honeybees and is composed mostly of glucose and fructose. However, it also contains vitamins, minerals, amino acids, enzymes, organic acids, and other compounds. Its composition is affected by seasonal variations as well as the geographic location where the nectar was gathered by the bees. The moisture content of the deposited nectar mixture reduces and dries out, becoming more concentrated and producing viscous honey [9,10,11,12].

Natural honey is composed of around 82% of water, carbohydrates, proteins, phytochemicals, antioxidants, and minerals. It has been proven that few of the ingredients that determine the biological and medical potential of this substance are likely to vary among the various types of honey [13]. The sugars in honey include, in descending order, the following: “fructose (38.2%), glucose (31.2%), disaccharides and some other tri-saccharides and higher saccharides (9%) and sucrose (0.7–1%)” [14]. Honey containing a wide range of active compounds, including flavonoids, organic acids, phenolic acid, vitamins, and enzymes, may improve wound healing [14]. The deposition of fibroblasts and collagen formation may also be promoted by the large amount of amino acids found in honey [15].

**Figure 1:**
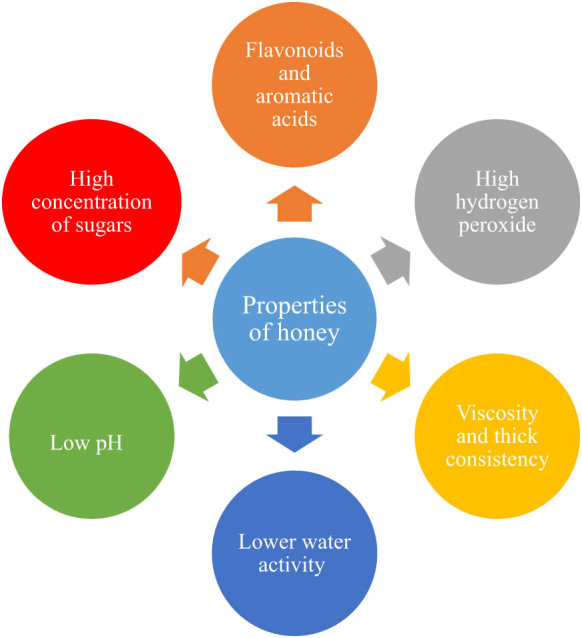
Some physicochemical properties of honey.

The natural properties of honey as well as its active compounds are crucial for the wound healing process (Figure 1). Natural honey is a viscous fluid; its jelly consistency creates a surface layer over the wound that inhibits the entrance of bacteria and protects the wound from dehydration [2]. Its high sugar content creates a higher osmotic gradient that pulls fluid up through the subdermal tissue and offers an additional glucose source for flourishing cellular components in the wounded area [2,16]. The water activity of honey is less than 0.91 aw, which prevents and controls the growth of bacteria on the wound surface [17,18] and causes fluid flow that flushes slough, debris, and necrotic tissue as well as microorganisms out of the wound. Apart from this, the low water activity of honey helps transport oxygen and nutrients from the deep tissue into the wound area. In addition, the low pH of honey increases tissue oxygenation, while free radicals, which lead to tissue damage, are removed by flavonoids and aromatic acids [19,20].

Another property that triggers antimicrobial activity in honey is the production of hydrogen peroxide on the glucose [5]. Certain types of honey do not rely on hydrogen peroxide for their antimicrobial activity but probably rely more on pH change and osmolarity for their bactericidal capability [5]. The unique Manuka factor (UMF) in Manuka honey (MH) is the methylglyoxal (MGO) level which is responsible for its antibacterial effect [21]. However, hydrogen peroxide-dependent honey stimulates the production of vascular endothelial growth factor (VEGF) and sterilizes the wound site [3,22]. In addition to glucose oxidase, the invertase produced by bees strengthens the osmotic potential of honey, dividing sucrose into fructose and glucose [3,22].

Two principal types of honey that have been researched are non-peroxidase MH and peroxidase based honey, both known for their efficacy in wound management [15]. Hence, the aim of this review is to summarize recent studies on the wound healing properties of honey.

## 2 The cascade of wound healing

The natural wound healing process includes a chain of events involving proteins, proteases, blood cells, growth factors, and ECM. The process comprises four successive and overlapping phases – hemostasis (blood vessels constrict to restrict blood flow), inflammation (controls bleeding and prevents infection), proliferation (rebuilding new tissue made up of ECM and collagen), and re-modeling (maturation) [23]. An imbalance in any one of these phases can result in over-induction of wound healing or attenuation of the healing process. In diseases, such as peripheral vascular diseases or type 2 diabetes, excessive inflammation can lead to a reduction or delay in the wound healing process [24]. On the other hand, over-induction of the healing process can be caused by excessive proliferation, resulting in scar or keloid formation.

## 3 Properties of honey

The therapeutic potency of honey is complex due to the presence of many compounds as well as variations in the composition of different types of honey [12,25]. It has specific physicochemical properties (Figure 1) that favor its use as a therapeutic agent to combat several microbial infections. These properties of honey are also associated with its wound healing effect, anti-inflammatory potency, antioxidant, and free radical scavenging ability. It is an immunomodulator with the power to enhance the immune system. It can be applied in the treatment of gastric ulcers, recurrent canine dermatitis, arthritis, diarrhea, tumors, and ulcers in diabetic patients; it can also be used for skin disinfection and wound healing [26,27]. In addition to its anti-inflammatory and antibacterial properties, honey enhances the wound healing process [7].

Honey is effective in curing a wide range of wound types, including trauma, burns, malignancy, leprosy, diabetic ulcers, boils, cervical varicose ulcers, scratches, leg ulcers, gastric ulcers, fistulas, amputation, burst abdominal wounds, septic and surgical wounds, cracked nipples, and wounds in the abdominal wall [28].

Many flavonoids, including pinobanksin, chrysin, and pinocembrin, as well as certain other compounds in lower levels, such as luteolin, quercetin, 8-methoxykaempferol, isorhamnetin, kaempferol, and galangin, are found in MH. Flavonoids provide honey with antioxidants and anti-inflammatory efficacy. Catechins, which are members of the flavone group of polyphenols, are often present in honey. Catechins have the potential to scavenge both superoxide and hydroxyl radicals [29] as well as the 1,1-diphenyl 1,3-picrylhydrazyl radical [30], proxy radicals [31], nitric oxide [32], carbon-centered free radicals, singlet oxygen and lipid free radicals [29], and also peroxynitrite by preventing the nitration of tyrosine [33].

## 4 Antimicrobial activities

According to the international guidelines on the proper use of antimicrobials in medicine, honey and other alternative therapeutics were used for the treatment of skin lesions in both humans and animals [34]. The antibacterial effect of honey has been reported in numerous studies [35,36,37]. Honey exerts bacteriostatic and bactericidal actions [26,37,38]. Many enzymes are present in an internal pouch of the bee called the crop and are transferred to the honey.

The antibacterial activity of non-peroxide honey is related to the presence of glyoxal, 3-deoxyglucosulose, and MGO. The concentration of MGO in honey is dependent on the geographic location and the kind of honey. However, it is well-known that MH has the highest concentrations of MGO compared to other types of honey. MGO is present in all kinds of honey, with levels ranging from 3 to 800 µg/gram, depending on the type of MH. The antibacterial efficacy of honey is dependent on the MGO content; honey will have a weaker or stronger effect on a narrower or wider spectrum of bacteria, particularly on the methicillin-resistant *Staphylococcus aureus* strains, vancomycin-resistant enterococci, and *Pseudomonas aeruginosa*. Nevertheless, studies have shown that a high MGO concentration is not required to exert antibacterial efficacy. For instance, in a study by Girma et al. (2019) [39], MH of lower UMF grade demonstrated significantly increased antimicrobial activity compared to higher UMF grade honey against tested *S. aureus* and *E. coli*. An MGO of 10+ UMF values were sufficient to provide antibacterial efficacy. It has been reported that high MGO may cause damage at the cellular level either through blood leading to its glycation or via other external pathways leading to malignant young cell degeneration [40].

The antimicrobial effects of honey have also been studied in various *in vivo* experiments, suggesting that this property of honey is crucial in reducing secondary bacterial contamination of the wound area and hastening the healing process [41].

Fluids in the wound are drawn out of the damaged tissues, leading to drying of cellular tissues and bacterial death [3]. In addition, phenolic compounds, organic acids, vitamins, and flavonoids exert antioxidant action and boost the antibacterial effect of honey. Flavonoids neutralize free radicals produced by hydrogen peroxide [3,22]. However, despite the increase in studies on the use of honey for wound healing, whether traumatic or surgical in origin, only a few studies on its use on infected wounds have been published. Some authors analyzed honey’s potential on the growth of selected intestinal bacteria [42] and in combatting pathogenic bacteria frequently isolated from skin wounds of mammals, including humans [35]. In a rat model, the topical application of honey on a dorsal wound resulted in an increase in both salt- and acid-soluble collagens by 107 and 117%, respectively. Further, it potentiated the levels of insoluble collagen to achieve a 109% increase after seven days of treatment compared to the untreated control [22].

Medical-grade honey (MGH) is seen as promising wound therapy because it has a wide spectrum of antimicrobial efficacy with no known resistant strains. It has been effective against clinical isolates of *Pseudomonas aeruginosa* and their associated biofilm formation [43].

Studies have shown that the supplements in the MGH formulation such as vitamins (C and E) enhanced the antimicrobial activity of pure honey. Supplementation of honey with other additives may, therefore, be a promising approach to further improve the antimicrobial activity of honey [44,45,46].

Another study evaluated the antibacterial efficacy of 57 Slovak blossom honeys against *Pseudomonas aeruginosa* and *Staphylococcus aureus*. Their data showed that different types of honey had different antibacterial potentials. Between acacia, wildflower and rapeseed honeys, the wildflower honey samples showed the greatest antibacterial activity, while rapeseed honeys had the highest level of minimal inhibitory concentration. There was a statistically significant association between the antibacterial activity of the honeys and their H_2_O_2_ and turgor pressure content. However, there was no correlation between glucose oxidase (GOX) and H_2_O_2_ content [47].

The results of another *in vitro* investigation revealed that lower supplemented honey at a lower percentage (40%) is more effective than 80% of MH against a wide range of commonly present cutaneous pathogens, including Methicillin-resistant Staphylococci and *Pseudomonas* species. Yet again, the supplements may have resulted in the improvement of the antimicrobial activity of honey [48].

## 5 MGH and its wound healing effects

Several types of honey, including MGH, have recently been re-introduced into modern medicine. There is no clear definition of MGH, but according to Hermann et al. (2020) [49], MGH must fulfill the following criteria:

- Purity and being organic.
- Free from toxic substances and contaminants.
- Sterilized by gamma radiation under standardized procedures and having no pathogenic microorganisms.
- Suitable for use in medical therapies.
- Adheres to standardized production and storage methods, and all legal and safety regulations;
- Satisfies physicochemical criteria necessary for use in wound treatment.

MH reduces inflammation and stimulates fibroblast migration and collagen deposition that support regeneration and hasten healing of the injured area [6]. Clinical studies have proven definitively that multiple varieties of honey, including MH, have the potential to close various types of infected non-healing ulcers [14,50,51,52]. In a study by Ranzato et al. (2012) [9], a 180% rate increase in keratinocyte closure and 150–240% fibroblast migration were recorded using 0.1% MH. Likewise, Efem [53] stated that application of honey on wounds caused rapid tissue debridement, stimulated quick epithelialization, and decreased the development of edema, causing quicker healing.

Bucekova et al. (2017) [54] reported that Def-1 peptide found in honey had a positive impact on cutaneous wound closure and exerted its effect by potentiating Keratinocyte migration and MMP-9 secretion.

MGH has attracted a great deal of interest recently; the term is used by healthcare professionals to refer to honey used in wound treatment [55]. In recent years, there has been a resurgence of interest in the use of MGH in the management of wounds. MGH possesses antibacterial efficacy and wound healing potential, including for mucositis in pediatric patients [26]. Some advantages of using MGH are that it is safe, easy to apply, and cost-effective for the treatment of severe wounds, burns, ulcers, oral mucositis in young pediatric patients, and in those suffering from cancer [56,57]. Honey’s anti-inflammatory effect and ability to treat local infections, promote autolytic debridement, disinfect wounds, and promote granulation tissue, which have been confirmed in previous literature [58]. A study evaluated the efficacy of honey in the treatment of wounds of lower leg and diabetic ulcers. The results showed a reduction in healing time, a much higher percentage of completely healed wounds, and better success in wound infections [50]. The therapeutic and wound healing effects of honey have been reported by others [59,60].

In a comparative study on topical application of MH and acacia honey in diabetic and normal rats, it was reported that MH achieved up to 80% wound contraction after nine days of treatment. In the MH-treated group, complete epithelialization was evident two days earlier than usual epithelialization [61].

The combined effect of topical honey with silver nanoparticles was assessed in an experimental wound healing process in rats, and the data showed that multiflora honey with silver nanoparticles enhanced the efficacy of wound contraction compared to honey alone [62].

The efficacy of topical application of mad honey, a rhododendron honey, was evaluated in wound healing in diabetic rats. The data showed that mad honey enhanced the healing process in diabetic rats and caused a significant decline in the levels of TNF-α, malondialdehyde, and MMP-9 expression. This was accompanied by an increase in the activities of the antioxidant enzyme and IL-10 expression in comparison to the untreated controls [63].

In addition, honey reduced excessive scar formation, thus improving the outcome of wound healing [64]. Based on that, the use of honey, which is a natural product, can be cost effective, safe, and efficient in the treatment of large and complicated wounds [65].

The mixture of bees’ honey and *N. sativa* is a relatively cheap and safe natural preparation used traditionally to cure human diseases since ancient times [66]. The combination enhanced the wound healing process by inhibiting the toxic effect of *N. sativa,* especially when the seed extract is used, in chronic and large surface area application [67].

Another study reported that the localized application of a mixture of honey and black seed oil in experimentally induced wounds on the ears of rabbits showed enhanced wound healing and a significant reduction in the wound area by the end of the fourth week. This finding suggests that the mixture of black seed oil and honey enhances wound healing without producing toxicity to the cells [68]. In a study on the synergistic effect of honey and *N. sativa* on wound healing was assessed in a rat model, and the results indicated that the mixture of honey and *N. sativa* seed oil significantly reduced the wound surface area compared to the control group [69].

The use of MGH as a therapeutic agent decreased malodor in a few days and stopped the infection within 2–3 weeks. MGH promoted wound healing by enhancing granulation tissue formation, angiogenesis, and re-epithelialization by reducing oxidative stress and providing nutrients.

Yilmaz and Aygin (2020) [70] conducted a systematic review in a randomized controlled study to evaluate the efficacy of honey in the wound treatment process. Their data showed that honey resulted in rapid epithelialization and wound contraction in wound healing, and reduced pain, inflammation, and debridement, ensuring control of infection and reducing the time of wound healing, and was cost-effective. The authors advanced the idea that MGH improved wound healing and patient’s quality of life. MGH is safe and cost-effective, especially when dealing with complicated diabetic wounds (antibiotic-resistant) with infections and the risk of amputation.

In their study, Smaropoulos and Cremers (2020) [57] reported the safety, efficacy, and usefulness of MGH in the treatment of abdominal wounds in pediatric patients. All treated wounds rapidly revealed granulation tissue formation and underwent re-epithelialization. There was a noticeable reduction in peripheral edema and inflammation upon initial application. An effective debriding of necrotic tissue was observed, and the same applied to sloughs, which were easily removed when detected with no sign of infection, regardless of initial wound presentation. There was minimal scarring with full preservation of movement in all cases.

In a systematic review carried out by Jull et al. (2015) [71] on the efficacy of honey in comparison with alternative wound dressings and topical treatment of acute burns, lacerations, and/or chronic wounds (e.g., venous ulcers), results suggested that honey healed partial thickness burns more quickly than conventional treatments (e.g., polyurethane film, paraffin gauze, soframycin-impregnated gauze, sterile linen, and leaving the burn exposed), and infection resulting from post-operative wounds healed faster than with antiseptics and gauze.

The results of a comparative study using topical applications of Acemannan gel (AG), hyaluronic acid, and MH on bilateral wounds introduced on the backs of six sheep indicated that treatment with AG resulted in wound dehydration and stimulated late granulation tissue and cell proliferation. Moreover, the AG-treated wounds had a mild late pro-inflammatory and neovascularization effect and a positive influence on moist wounds with abundant granulation tissue and exudate [72]. In contrast, the MH-treated wounds were slightly dry. The main effect of MH was to promote cell proliferation and neovascularization, with an overall pro-inflammatory effect. Results suggest that MH treatment enhanced the healing process [72].

Table 1 summarizes the effects of different types of honey, including Manuka honey, in acute, chronic, and mixed types of malignant wounds.

**Table 1:** Effects of various types of honey on classes of wound

Wound class	Wound type	Type of honey	Effect of honey	References
Acute	Burns	Manuka	Control of infection and inflammation, shorter healing time	[73]
	Surgical and traumatic	Indian *Syzygium cumini*	No significant difference	[74]
Chronic	Infected, surgical and traumatic	Manuka	Significant clinical improvement	[75]
	Pressure ulcers	Medihoney	Shorter healing time	[76]
	Lower extremity ulcers	Pakistani Beri (*Ziziphus jujuba)-*honey	Shorter median healing time	[77]
Mixed acute and chronic	—	Medihoney^TM^	Significant decrease in wound size, perceived pain levels, and wound sloughing/necrosis	[78]
Malignant	—	Manuka honey	Decreased odor and inflammation	[79]

## 6 Underlying mechanisms of honey in wound healing

The antibacterial activity of honey has been well documented [35,36,37,80]. Honey activity can be either bacteriostatic or bactericidal, depending on the kind of honey [37]. The internal pouch of the honeybee known as the crop is considered the reservoir for many enzymes that are added to honey.

**Figure 2:**
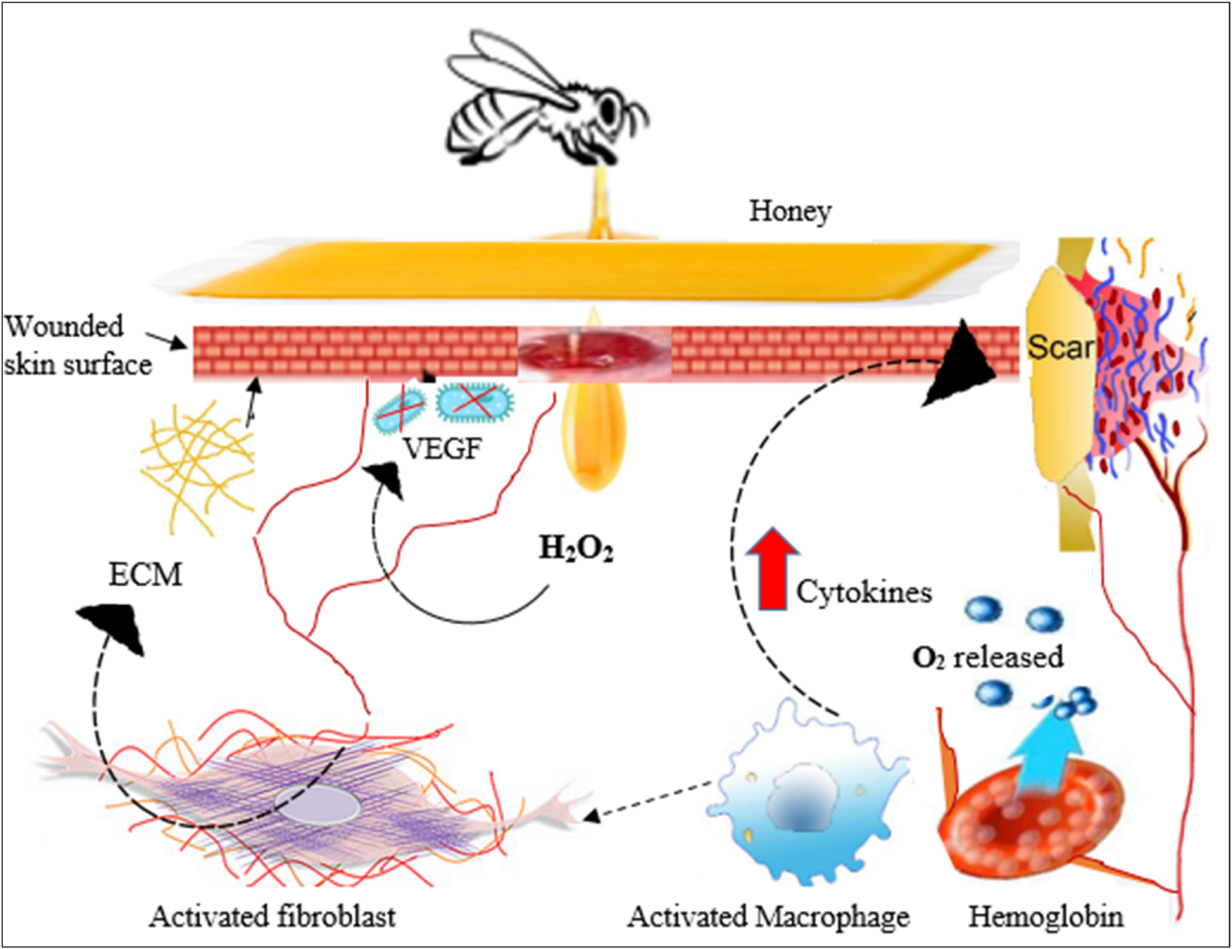
Antibacterial and wound healing effects of honey (ECM, extracellular matrix; H_2_O_2_, hydrogen peroxide; VEGF, vascular endothelial growth factor; O_2_, oxygen).

One of these enzymes is glucose oxidase, which catalyzes glucose oxidation to form gluconic acid and hydrogen peroxide. The production of gluconic acid results in lowering the pH, and hydrogen peroxide enhances its bactericidal efficacy [3,22]. Therefore, to lower the pH levels between 3.5–4, a series of events essential for the process of tissue repair takes place: reduction in protease activity in the wound site, increase in the oxygen release from hemoglobin, and stimulation of fibroblast and macrophage activity. Furthermore, the production of hydrogen peroxide stimulates VEGF and sterilizes the wound (Figure 2) [3,22]. Another important enzyme produced by the honeybee is invertase, which provides the honey with stronger osmotic potential by hydrolyzing sucrose into fructose and glucose [3,22]. Fluids in the wound are oozed out of damaged tissues, leading to drying of cellular tissues and bacterial death [3].

Notably, phenolic compounds, organic acids, vitamins, and flavonoids exert antioxidant activities and boost the antimicrobial effect of honey. Flavonoids neutralize free radicals produced by the hydrogen peroxide [3,22]. In addition, the immunomodulatory effects of honey enhance wound healing, and various ingredients in honey contribute to its anti-inflammatory and antioxidant properties [81]. Moreover, the high concentration of nutrients promotes epithelialization and angiogenesis [2]. An important source of nutrients for the tissues is derived from the presence of carbohydrates, especially glucose and fructose, with maltose, sucrose, and isomaltose in smaller quantities. Carbohydrates represent about 80% of honey’s components [22,82,83,84]. Certain types of honey exert their bactericidal efficacy primarily by bee defensin-1 and the glucose oxidase enzyme. The latter enzyme changes honey sugar into gluconic acid and 3% of hydrogen peroxide, tolerated by tissues and effective against bacteria [85].

## 7 Future directions with tissue-engineered honey-impregnated scaffolds

Despite the increase in studies on the use of honey for wound healing, whether of traumatic or surgical origin, only a few studies on its use on infected wounds have been done. Some authors have analyzed honey’s potential on the growth of selected intestinal bacteria [42] and against pathologic bacteria frequently isolated from the skin wounds of mammals, including humans [35].

Nanotechnology is an emerging field that has found its way into many applications, including medicine, drug delivery, and cosmetics, and its application in medicine is growing very rapidly [86]. In the past decade, tissue has been widely investigated. Honey initially used in wound healing was an 80–100% MH and a gelling agent; however, due to its high osmolarity, it tended to leak out of the bed of the wound. To resolve this issue, scaffolds were implemented to provide slow and controllable release of honey to maintain the absorption of wound exudate (Table 2). Currently, tissue-engineered honey-infused scaffolds include mostly electrospun fibers, cryogels, and hydrogels. Such scaffolds may provide a better honey delivery system [7,87]. In addition, several presented clinical cases illustrated that honey formulation comprising natural wound care products such as L-Mesitran Ointment, L-Mesitran Soft, and the associated ingredients (vitamins, polyethylene glycol, etc.) enhance the healing properties of honey [43].

**Table 2:** Currently researched types of honey-infused scaffolds

No.	Techniques	Characteristics	Drawbacks	References
1	Electrospinning	Manufactured by impregnating honey into electrospun nanofibers. High surface area-to-volume ratio enables bio-resorption and a permeable structure. Considered easiest to apply, most efficient, and cost‐effective	Fiber morphology can be affected by the addition of honey	[7]
2	Hydrogels	A solid gel-like structure made from a polymer solution cross-linked at ambient temperature	Many types of honey and polymers are used to produce these hydrogels	[7]
3	Cryogels	Produced by freezing a polymer solution immediately after cross-linking, causing ice crystals to form surrounded by the formation of gel-like matrices	Such scaffolds have a wide range of active ingredients, making it difficult to evaluate the efficacy of individual honey types	[88]

## 8 Conclusion

MGH is a promising wound healing agent because it has a broad spectrum of antimicrobial efficacy with no known resistant pathogens. It has been shown to be effective against clinical bacterial and fungal isolates and their associated biofilm formation in a dose-dependent manner. It is safe and cost-effective, especially in the treatment of different types of wounds. MGH should be considered a potential alternative to antibiotics or complementary therapy for treating locally infected wounds. An improved delivery system and a structure to support wound healing could tremendously enhance the treatment process and result in better outcomes.
